# Carbohydrates Metabolic Signatures in Immune Cells: Response to Infection

**DOI:** 10.3389/fimmu.2022.912899

**Published:** 2022-07-04

**Authors:** Kareem Awad, Amany Sayed Maghraby, Dina Nadeem Abd-Elshafy, Mahmoud Mohamed Bahgat

**Affiliations:** ^1^ Department of Therapeutic Chemistry, Institute of Pharmaceutical and Drug Industries Research, National Research Center, Cairo, Egypt; ^2^ Research Group Immune- and Bio-Markers for Infection, the Center of Excellence for Advanced Sciences, National Research Center, Cairo, Egypt; ^3^ Department of Water Pollution Research, Institute of Environmental Research, National Research Center, Cairo, Egypt

**Keywords:** carbohydrate metabolism, immune cells, pathogens, COVID-19, glucose

## Abstract

**Introduction:**

Metabolic reprogramming in immune cells is diverse and distinctive in terms of complexity and flexibility in response to heterogeneous pathogenic stimuli. We studied the carbohydrate metabolic changes in immune cells in different types of infectious diseases. This could help build reasonable strategies when understanding the diagnostics, prognostics, and biological relevance of immune cells under alternative metabolic burdens.

**Methods:**

Search and analysis were conducted on published peer-reviewed papers on immune cell metabolism of a single pathogen infection from the four known types (bacteria, fungi, parasites, and viruses). Out of the 131 selected papers based on the PIC algorithm (pathogen type/immune cell/carbohydrate metabolism), 30 explored immune cell metabolic changes in well-studied bacterial infections, 17 were on fungal infections of known medical importance, and 12 and 57 were on parasitic and viral infections, respectively.

**Results and Discussion:**

While carbohydrate metabolism in immune cells is signaled by glycolytic shift during a bacterial or viral infection, it is widely evident that effector surface proteins are expressed on the surface of parasites and fungi to modulate metabolism in these cells.

**Conclusions:**

Carbohydrate metabolism in immune cells can be categorized according to the pathogen or the disease type. Accordingly, this classification can be used to adopt new strategies in disease diagnosis and treatment.

## 1 Introduction

### 1.1 Metabolic Pathways in Immunocytes

Immune cells are flexible in terms of their ability to dynamically reprogram their cellular metabolism. This flexibility can overcome diverse conditions of nutrients and oxygen availability in multiple tissues and contain significant metabolic stresses from their micro-environmental conditions (1– 2). A clear example is a lymphocyte that can turn from a relatively static cell to another involved in growth and proliferation that produces considerable cytokine storms or effector molecules (3). Moreover, metabolic programming will also affect specific levels of metabolites that consequently result in different immune cell functionalities (1, 4, 5). These changes in metabolite levels include a rich and diverse set of post-translational modified forms of enzymes or products of glycolysis and tricarboxylic acid (TCA) cycle in immune cells that have not been fully understood yet (6). These modified enzymes or products result in different alterations of immune cell phenotypes and consequently, modulate immune responses and inflammation in different contexts (6). Beyond the utilization of specific metabolic pathways, metabolic reprogramming in immune cells also repurposes their oxidation–reduction system, as shown through the role of reactive oxygen species (ROS) in the proliferation and functionality of cytotoxic and humoral immune cells as well as monocytes and macrophages (MQs) (7).

The immune system encompasses populations of various cells that are substantially dynamic in terms of their proliferation, survival, and functions in response to different immunological stimuli and share the ability to rapidly respond to infection, trauma, and other perturbations (1, 3). The need for energy of such cellular functions is essential with glucose metabolism being the major carbon fuel source through two distinct pathways: glycolysis as a first pathway producing pyruvate and adenosine triphosphate (ATP) and TCA as an immediately following pathway (4, 8). Metabolic reprogramming mainly happens through specific receptors and the tuning of growth-factor cytokine signaling, as well as nutrients and neuronal regulation that are all dynamic factors connected to the existence of a pathogen (9, 10).

Pathogens such as bacteria, fungi, parasites, and viruses have developed sophisticated tools to manipulate their victims’ cells for their proliferation and existence, so the normal structures and functions of essential mammalian host organelles such as endosomes, Golgi apparatus, mitochondria or even their membrane components or nuclear envelopes are targeted by microbial machines (11). Here, we analyzed how the immune cell metabolism is manipulated by pathogens including bacteria, fungi, parasites, and viruses, with a focus on the interaction with specific immune cell metabolic pathways.

### 1.2 Methodological Approaches Used to Identify Metabolites in Living Cells

Metabolomic analysis of infected cells provides insights to the formation and consequences of the common metabolic pathway changes (12). These include metabolites essential for glycolysis, amino acid and fatty acid synthesis, and uncommon metabolic changes including those using xenobiotics such as the sugar substitute erythritol and chemicals like phenol red (12).

Pathogens exert particular mechanisms to alter metabolic pathways to continue their life cycle and such mechanisms represent candidate therapeutic targets to treat infections (12–14). Metabolomic approaches are widely used to study changes in cellular metabolism (15–18) among which is the use of magnetic resonance spectroscopy, mass spectrometry, or gas and liquid chromatography to concomitantly detect various metabolite alterations (15–18). These approaches have widely allowed for the determination of infections or tumor-induced alterations in metabolism. For example, the levels of metabolites involved in glycolysis and Krebs cycle remarkably increased as detected by liquid chromatography-tandem mass spectrometry when the human cytomegalovirus (HCMV) progresses into an infection in human fibroblasts (15).

Similar metabolomic approaches, as well as these metabolites, have been used to identify novel proteins and lipid molecules during hepatitis C virus (HCV) infection in cultured cells (18). These included two fatty-acid oxidation enzymes hydroxyacyl-CoA dehydrogenase beta subunit (HADHB) and dodecenoyl coenzyme A delta isomerase that have demonstrated differential regulation both *in vitro* and in HCV-infected patients with coincidence with cytopathic effect in cell culture and significant liver lesion in HCV infected patients (18). *In vitro* analysis by ^1^H NMR and then ^31^P NMR of extracts from malignant tumors indicated an initial reduction in phosphocholine, glycerophosphocholine, and myo-inositol, and then phosphoethanolamine and glycerophosphoethanolamine, respectively (17). In keeping with these results, as measured by ^1^H NMR, inhibition of the hypoxia-induced factor 1 (HIF-1) with PX-478 as an experimental anticancer agent gave rise to a considerable change in total choline *in vivo* (17).

Here, we summarized the changes in carbohydrate metabolomic immune cells associating infections with various pathogens with the aim to identify pathogen/disease-specific carbohydrate metabolic immune cell signatures of potential diagnostic and/or prognostic relevance.

## 2 Methods

**Figure 1:**
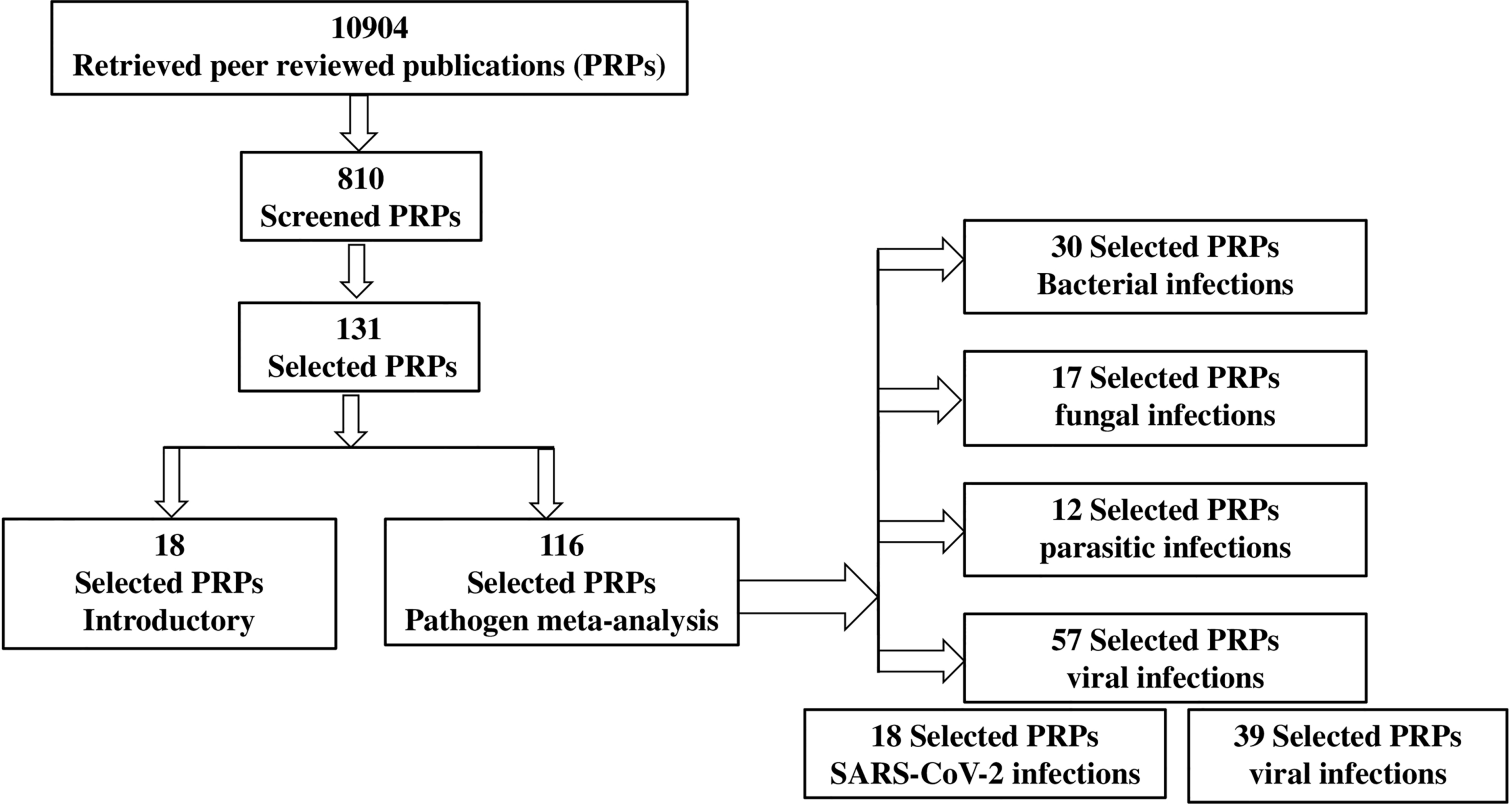
PRISMA flow diagram of the systematic review.

A literature search of medical research electronic databases was conducted for peer-reviewed articles (**Figure 1**). These included Medline (PubMed), produced by the National Library of Medicine, Washington, USA; Embase (Scopus), produced by Elsevier, Amsterdam, the Netherlands; the Cochrane Central trials register, produced by John Wiley & Sons, Ltd.; and Clarivate, produced by Thomson Reuters, USA. This research was done in the time period 2018–2022. The key search terms used were T cell, monocyte MQ, immune, CD^+^4 –T, inflammation immune cell,NK, and innate and adaptive immune cell metabolism in combination with various pathogens including bacteria, fungi, parasites (including protozoa and helminths), viruses, and COVID-19.

Titles and abstracts were carefully screened and full-text peer-reviewed published articles were retrieved. Data were extracted based on the PIC algorithm (pathogen type/immune cell/carbohydrate metabolism). Accordingly, we subtracted information from studies on single infections in one immune cell model while those with multiple infections were excluded. Also, publications on sugar metabolic pathways only in immune cells were included, while other metabolic pathways in either somatic cells or immune cells were excluded, unless exceptionally showing relation to glucose metabolism.

Out of the 131 reviewed papers, 30, 17, 12, and 57 have explored immune cell metabolic changes accompanying bacterial, fungal, parasitic, and viral infections, respectively. Current perspectives on immune cell specific metabolism due to the current COVID-19 pandemic were from 18 publications out of the total 57 manuscripts related to viruses (**Table 1**). A total of 18 reviewed papers introduced and defined immunometabolism and the scientific methods used in its study. The reviewed bacterial pathogens were *Legionella pneumophila*, Mycobacterium species, *Pseudomonas aeruginosa*, and *Salmonella enterica*. The reviewed fungi were *Candida albicans* and entomopathogenic fungi (EPF). The included protozoa parasites were *Entamoeba histolytica*, *Theileria annulata*, and *Toxoplasma gondii*, whereas the helminth parasites were *Fasciola hepatica* and *Schistosoma mansoni*. The reviewed viruses were DNA viruses such as adenovirus E4ORF, varicella-zoster virus (VZV), Epstein–Barr virus (EBV), and HCMV. RNA viruses such as coxsackie virus B3, dengue virus (DENV), human immunodeficiency virus (HIV), HCV, poliovirus, influenza virus, yellow fever, and West Nile were also discussed. Virus-induced cytokines were briefly argued.

**Table 1:** Number of analyzed articles and reviewed diseases in this review.

Number of articles used for the analysis of immune cell metabolism during bacterial infection	30
**Number of bacteria** **Names of bacteria**	5
	*Legionella pneumophila* *Mycobacterium tuberculosis* *M. bovis* *Pseudomonas aeruginosa* *Salmonella enterica*
**Number of articles used for the analysis of immune cell metabolism during fungal infection**	17
**Number of fungi** **Names of fungi**	1+
	*Candida albicans* Entomopathogenic fungi, EPF
**Number of articles used for the analysis of immune cell metabolism during parasitic infection**	12
**Number of parasites** **Names of parasites**	5
	*Entamoeba histolytica* *Theileria annulata* *Toxoplasma gondii* *Fasciola hepatica* *Schistosoma mansoni*
**Number of articles used for the analysis of immune cell metabolism during viral infection**	57
**Number of viruses** **Names of viruses**	13
	Adenovirus E4ORF1 Epstein–Barr virus (EBV) Human cytomegalovirus (HCMV) Varicella-zoster virus (VZV) Coxsackievirus B3 DENV Hepatitis C virus (HCV) Human immunodeficiency virus (HIV) Influenza virus Poliovirus Yellow fever virus West Nile virus SARS-CoV-2

## 3 Results

### 3.1 Bacteria

The effect of bacterial pathogens on the infected host’s metabolism had been long identified in animals and plants (19–21); however, understanding bacterial modifications, particularly of immune cell metabolism, can be important in developing host-directed immune therapies. This drives current attention to the analysis of bacterial-induced metabolites, which manipulate immune cell proliferation, differentiation, and function.

**Figure 2:**
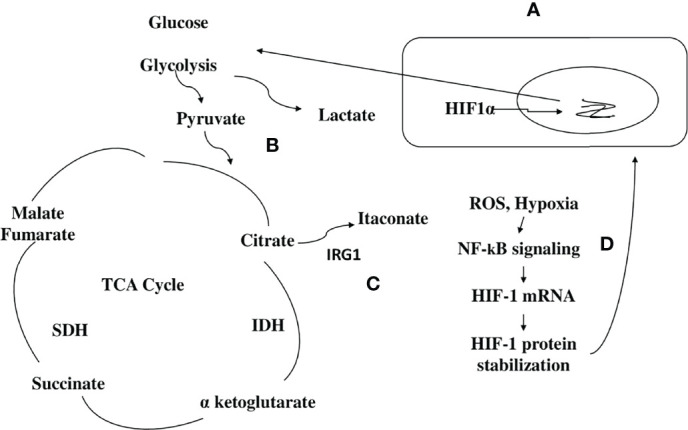
Scheme hypothesizing how itaconate regulates TCA flow in monocytes/macrophages infected with a bacterial pathogen. (1) **(A)** During bacterial infection, activated macrophages (MQs) accumulate stabilized HIF-1α, which subsequently translocates to the nucleus, initiating the transcription of genes related to glycolysis. **(B)** Activated MQs and other surrounding immune cells such as induced T cells have shown high glycolysis during infection that is accompanied by Warburg-effect metabolism (accumulation of lactate). **(C)** Immune responsive gene 1 (IRG1) links cellular metabolism with immune defense by catalyzing itaconic acid production in mouse and human macrophages, and itaconate modulates MQ metabolism and effector functions by inhibiting succinate dehydrogenase (SDH)-mediated oxidation of succinate and thus, controls succinate levels and function. **(D)** Instead of producing ATP, this metabolic switch repurposes succinate oxidation in the mitochondria to release bactericidal ROS catalyzed by SDH and isocitrate dehydrogenase (IDH). An organism like *Pseudomonas aeruginosa* can also sense ROS, which promotes an acquired change, the biofilm formation.

A frequent cause of severe pneumonia in humans is *Legionella pneumophila*, which is used as a model for exploring immune responses to intravacuolar bacteria (22). Remodeling of the TCA cycle is a metabolic adaptation during MQ polarization as recently shown for succinate, which regulates the pro-inflammatory IL-1β- hypoxia-inducible factor-1α (HIF-1α) axis (23). During *L. pneumophila* infection, type I and II interferons (IFNs) are master regulators of gene expression and stimulators of alveolar MQ-intrinsic immune responses that inhibit bacterial growth during the course of the disease (22). Type I or II IFN-dependent production of immune responsive gene 1, the mediator of the bactericidal itaconic acid, has shown control on infection in MQ and also inhibits pro-inflammatory cytokine expression and mitochondrial respiration (22–24). In both human and mouse immune cells, itaconate modulates the MQ metabolism and effector functions by inhibiting the succinate dehydrogenase (SDH)-mediated oxidation and thus, controlling the succinate levels and function (23–25). So, during *L. pneumophila* infection, mitochondrial respiration can be modulated during MQ activation because of the change of itaconate levels (**Figure 2**).

Tuberculosis (TB) granulomas are agglomerations of induced and uninduced MQs, neutrophils, T cells, and other immune cells (26). Several unique metabolic modifications occur to modulate the behavior of immune cells within the granuloma, potentially boosting bacterial persistence against immunopathology (26). Growth inhibition has been demonstrated in *Mycobacterium tuberculosis*, expressing isocitrate lyase bacteria by itaconic acid in human and mouse MQs that is significantly reversed by Irg1 gene silencing (25). This suggests that IRG1 links cellular metabolism with immune defense by catalyzing itaconic acid production in mouse and human MQs (**Figure 2**) (25).

A unique opportunity to study the influence of a bacterial infection on glucose metabolism within immune cells has been provided with the involvement of many metabolically active immune cells during different stages of TB infection (27). Apparently, what characterize cancer cells and virus-infected cells are not far from what might also be called bacterial Warburg-effect type of metabolism, which highly dominates during TB infection among different immune cells (25). Activated M1 MQ and other surrounding immune cells such as induced T cells have shown high glycolysis during TB infection that is accompanied by Warburg-effect metabolism due to the accumulation of imported glucose (27).

High-resolution nuclear magnetic resonance spectroscopy detected an increase in lactate as TB progressed in the lungs of infected guinea pig evidencing the pathway of glycolysis during* *infection (28). Lactate detection is an indicator for the Warburg-effect metabolism in cancer or low oxygen demand metabolism type (29). Lactate release is common in mycobacterial infection across species as relatively high amounts of lactate have been detected in the serum of *M.* *bovis* and *M. tuberculosis*-infected mice and cattle (30, 31). Lactate activates an MQ phenotype characterized by increased activity of arginase 1 (32) that was hypothesized to favor nitric oxide (NO) inhibition, a key antimicrobial response. Substantially, lactate metabolism and signaling would be valuable host-directed therapy targets for TB treatment; however, crucial scientific effort is still needed to better understand the role of lactate in the host–pathogen interaction throughout the TB infection (33, 34).

A lesson is introduced by the *Drosophila melanogaster*, that is, short-chain fatty acids produced by fermentation of bacterial carbohydrates and other bacterial metabolites are sensed by specific G-protein surface receptors of enteroendocrine cells and enterocytes, or captured and converted by enterocytes into metabolically active molecules such as acetyl-CoA (35, 36). This modulates the host carbohydrate and lipid utilization systemically in the intestine by stimulating the release of enteroendocrine peptides into the systemic circulation that activates other signaling cascades (35, 36).

The bacterial pathogen *Pseudomonas aeruginosa*, which causes cystic fibrosis, exploits metabolic elements such as succinate and itaconate, to induce metabolic and transcriptomic modulations in immune cells that increase its capacity to cause stubborn diseases (37). *P. aeruginosa*, through the process of catabolite repression, preferentially consumes succinate compared to other carbon sources (38). Metabolic reprogramming in activated MQs switches to aerobic glycolysis instead of oxidative phosphorylation pathways in the mitochondria (39). Instead of producing ATP, this metabolic switch repurposes succinate oxidation in the mitochondria to release bactericidal ROS catalyzed by SDH and isocitrate dehydrogenase (IDH) (**Figure 2**) (39).

This enzymatic reaction is potentiated by a biochemical process called anaplerosis that favors the accumulation and synthesis of succinates from foreign metabolites, such as environmental glutamine (39, 40). Thus, *P. aeruginosa* provides itself with a favored substrate, succinate. Excess succinate is with time, compensated by the TCA cycle intermediate, itaconate, that inhibits MQ SDH and blocks glycolysis by altering the enzymatic function of both glyceraldehyde 3-phosphate dehydrogenase and aldolase (41, 42). Itaconate, in this way, functions as a major immunoregulatory molecule that resolves inflammation by modulating MQ metabolism. In this context, early infection by *P. aeruginosa* that is accompanied by succinate increase compared to a late or chronic infection that is accompanied by itaconate compensation should be considered when designing therapeutic strategies for this infection.

In another considerable mechanism, *P. aeruginosa* alters its immunometabolism in response to itaconate, which induces bacterial membrane stress, resulting in the upregulation of extracellular polysaccharides (EPS) and downregulation of lipopolysaccharides (LPS) (43). This process favors itaconate assimilation and the formation of a biofilm on the one side and on the other side, EPS, inducing itaconate production by myeloid cells and skewing the host immune response to one permissive chronic infection (43).

Another modulation of immunometabolism by *P. aeruginosa* is that it requires glucose transport *via* glucose transporter 1 (Glut1) in MQ as demonstrated in a sepsis mouse model where increased glucose uptake in MQs post-infection and lipopolysaccharide (LPS) production have been shown (44, 45). The activated serine-threonine protein kinase Akt1 which is essential for salmonella infection of MQs, phosphorylates the E3 ligase Mdm2, which ubiquitinates p53 and thus, may lead to increased glucose flux (46).

Although immunometabolism alterations in response to bacterial infection show a similar trend in experiments done in mouse and human cells, differences have been reported in comparable studies (25, 47). Intracellular itaconic acid concentration has demonstrated almost double production in mouse immune cells compared to human primary MQs (25). Another difference between mouse and human immune cells is the production of NO in mouse MQs that increases inflammatory conditions (47). NO inhibits aconitase, the enzyme producing the itaconic acid precursor *cis*-aconitate (48).

### 3.2 Fungi

Fungal effectors have often been recognized for their diversity, while current research has focused on effector biology, highlighting that different fungi utilize similar mechanisms for creating a more harmonious host environment, among which is manipulating the host immune metabolism (49, 50). Host immune responses have been suppressed by bioactive metabolites from fungi that act as immune modulators (49, 50). When considering fungi of human medical importance such as *Candida albicans*, different proteomic methods have detected enzymes at the cell surface of this fungus that are involved in cell wall glycan biosynthesis (for example, Uridine diphosphate (UDP)–glucose pyrophosphorylase) and may show immune cell modulation (51, 52). It is worth mentioning that *C. albicans*, in low glucose conditions, effectively utilizes various fermentable and non-fermentable carbon sources to remain and grow. Utilization of alternative carbon sources highly impacts the need to understand all these pathways as potential and attractive curative host targets (53).

**Figure 3:**
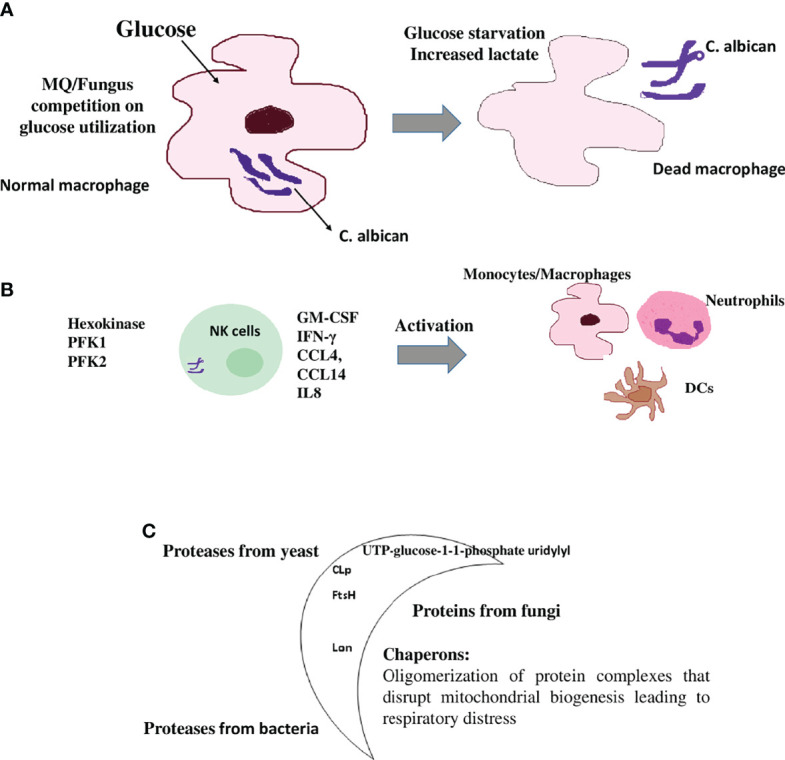
Immunometabolic changes in immune cells upon fungal and parasitic infection **(A)** MQs are metabolically inflexible and depend on glucose as a carbon source. The discussed *Candida albicans* fungus switches from yeast to filamentous hyphae, once taken by MQs. Fungal hyphae reactivate glycolysis and compete with MQs for glucose, which leads to depletion of glucose available to this immune cell by the fungus. MQ is killed by glucose starvation (56). High glycolysis is a metabolic shift in MQs during *C. albicans* infection. High glycolysis also results in increased lactate (Warburg-effect metabolism status) that is also utilized by *C. albicans*. **(B)** Natural killer (NK) cells respond to *C. albicans* pathogenII with the upregulation of genes involved in immune signaling and release of inflammatory cytokines that are involved in the activation of other immune cells as well as a shift to aerobic glycolysis. These include IFN-γ, GM-CSF, CCL4 (MIP-1β), IL8 (involved in the recruitment and activation of neutrophils), CCL14 (monocytes activator), XCL1 (dendritic cells activator), and the glycolytic enzymes hexokinase 2, phosphofructokinase1 (PFK1), phosphofructokinase fructose-2,6-bisphosphate (PFK2), and lactatedehydrogenase. **(C)** The moonlighting proteins: multifunctional proteins act as chaperones and are widely involved during the pathogenesis of infection through cell surface presentation. Some pathogenic bioactive metabolites are immune modulators and have been implicated in suppressing the host immune response. For example, in bacteria, the production of FtsH and CLp proteases, which are responsible for infectivity and virulence of a number of bacterial pathogens. In *C. albicans*, UTP-glucose-1-1-phosphate uridylyl transferase, an enzyme involved in cell wall glycans biosynthesis, has been detected at the cell surface of this fungus and shows immune cell modulation. In helminths and protozoan parasites, the synthesis of glycoconjugates and glycan-binding proteins such as the mitochondrial Lon protease are examples of these immune modulators.

Metabolic reprogramming has also been shown in the *C. albicans* infection of monocytes/MQs as killed hyphae and yeast induce gene expression that regulates the glycolytic pathway to a higher extent (**Figure 3A**) (54–56). Activation of MQ using *C. albicans* yeasts led to an increase in cellular respiration compared with the LPS/IFNγ-activation of MQs (57). *C. albicans* also* *reprograms NK cells toward higher glycolysis, which is required for proper lytic function of this cell type (**Figure 3B**) (58). This metabolic shift in immune cells toward higher glycolysis by *C. albicans* associated this infection to developed point mutations and metastasis in several studies (59, 60). It is noteworthy that the increase in glycolysis and glycolytic gene expression such as phosphofructokinase and pyruvate kinase were reported to have been highly expressed in MQs of both infected mouse and human cells (61, 62). Although high glycolysis could be needed by *C. albicans* to proliferate and escape immune mechanisms in the host, it also results in increased lactate that is thought to be an important carbon source for *C. albicans* in humans, where it is produced by activated inflammatory MQs that have switched to Warburg-effect metabolism (**Figure 3A**) (63, 64).

Modulating innate and humoral responses is crucial for EPF; yet, any manipulation that is detrimental to the host could result in displacement of EPF and opportunistic microbes causing sepsis (49). Immune modulators could burden the host’s guards and facilitate fungal colonization and biomass generation with the production of copious conidia needed for propagation and proliferation (65). Suppressing the host immune machinery saves the EPF’s required energy for the sake of colonization and growth rather than containing the host immune responses as shown in both human and mouse cells (8). Note that many known human immune suppressant medicines such as myriocin and cyclosporine originated from bioactive EPF (8). However, studies that can show the role of these metabolites in modulating metabolism in immune cells are lacking.

### 3.3 Parasites

Parasites are larger organisms that secrete metabolites able to interact and modulate immune cell metabolism in both human and animal cell models, while taking in account the mutual signaling among the complex network of parasites including two or more hosts (66–68). Despite their complexity, they depend on their hosts that often, for long periods, supply a rich environment that supports their growth, maturation, and reproduction (66–69). The protozoan parasite *E. histolytica* adopts strategies for immune evasion including suppression of IFN-γ production and other immunomodulatory mechanisms (66, 69). Reported metabolic alterations include the elimination of immune cells and other immune modulators as well as reactive nitrogen and oxygen species to overcome the attack from the immune system (66, 69). On the other hand, the protozoan parasite *T. gondii* can oppose genetic repression of its glucose transporter by increasing glutamine-derived carbon flux through the Krebs cycle and by synchronously activating gluconeogenesis, producing enough ATP required for the parasite replication and host-cell invasion (68).

As multicellular parasites, helminths promptly associate with their cell surface to the human host surroundings of various host ligand proteins such as plasma or extracellular matrix proteins as well as blood and immune cells (67, 68). An example of a complex biological matrix component that is constantly exposed to the host immune system is the outer tegumental coat of *F. hepatica* (FhTeg), a multicellular helminth that causes fasciolosis in humans (68). This FhTeg consists of glycolytic motifs and active glycoproteins that modulate immune cells (67). Among these proteins are a number of immune modulators such as tetraspanin CD63 membrane surface receptor, which is involved in the innate immune cell polarization, differentiation, and adhesion (70). Another molecule is TRIL (TLR4 interactor with leucine-rich repeats) that can bind to LPS and could hinder LPS activation of mast cells and dendritic cells (DCs) (50, 51). *F. hepatica* nardilysin-like molecules may associate with T-cell imbalance or prevent the activation and binding of TNFα-converting enzyme (71). *S. mansoni* is another helminth parasite that secretes components that can condition immune cells such as omega-1, a glycosylated T2 ribonuclease (Rnase) that conditions DCs to prime T helper 2 responses (68).

The common feature of helminths and protozoan parasites to respond to and counteract changes in their macro- and micro-environments, as well as to synthesize glycan-binding proteins and glycoconjugates for their protection, is broadly involved during the pathogenesis of infection through cell surface presentation of the so-called moonlighting proteins (72). The divergent extensiveness of these proteins in almost all organisms raises a question about the reason for their existence. One elucidation is the multifunctionality of these proteins. For instance, human lysyl hydroxylase 3 is an enzyme that catalyzes a few stages in the metabolic pathway and is also responsible for three consecutive steps through hydroxylysine-linked carbohydrate formation in post-translational modifications of collagen (73).

Another example is the economic usage of the mitochondrial Lon protease as one molecule, which, based on the environmental status and the needs of the cell, converts between chaperone functions and protein degradation (74). This phenomenon could have evolved during the formation of unique protein organizations that happened without changes to previous primary functions or structures of the questioned protein, or through the adaptability of the surface of unused molecules as new catalytic or binding sites for other diverse ligands (75). These multifunctional proteins could have been preserved during the course of cell evolution, as long as this alternative function does not obstruct the original enzymatic function (**Figure 3C**) (72, 75).

Direct interaction between parasites and metabolic behaviors of specific immune cells has been rarely studied in extracted cells from animal models (76, 77). The apicomplexan parasite *T. annulata* infection of MQ cell lines in normoxic conditions has driven its host cell to perform Warburg effect-like glycolysis which has been shown to be HIF-1α-dependent and is suggested as a way to overcome toxic levels of oxidative stress that originated from a *Theileria* infection and then resulted to uncontrolled proliferation of the host cell (76). In a separate study, IL-10, which is produced by MQ in various immune occasions and is important in controlling immune-mediated pathology, is produced by murine MQ derived from the bone marrow and exposed to *S. mansoni* cercariae secretory/excretory products in a mechanism linked to altered glycolysis, Krebs cycle, and oxidative phosphorylation and is involved the production of TLR2 and TLR4. However, there are concerns on the increase in glucose uptake and glycolytic kinase activity, and not the production of lactate (77).

### 3.4 Viruses

Metabolic alterations caused by virus infections are not limited to the infected cells but rather expand to other host cell types that respond to the infection and impact both the pattern of human and experimental animal viral diseases (78). These metabolic changes are necessary for tasks leading to virus replication, a process, which in a very short time, demands a considerable amount of energy (78, 79). Experiments on different viruses have demonstrated species- and time-related infected cells’ metabolic patterns obviously because of the different productivities when it comes to virus-type specificity or the infection dynamics reflected in the host cell type-specific metabolism (2, 5, 80–82). Moreover, there have been discussions on the changes in metabolic dynamics in different human and mouse immune cells on the infection with Epstein–Barr virus (EBV), flu, and human immune deficiency virus (HIV) and are reported with glycolytic activity, for example, with the metabolite phosphoenolpyruvate (PEP) activity and the intracellular redox state (82). It has been shown that the different immune cells derived from human or animal subjects express different metabolic patterns such as in the reduced activity of the TCA cycle in MQs and DCs, higher glycolysis in M1 MQs, TCA cycle-based M2 MQ metabolism, decreased oxidative phosphorylation activity in monocyte-derived DCs, and the central role of the mechanistic target of rapamycin (mTOR) in natural killer (NK) cells (83). These patterns can be shifted, relying on the new human body’s status observed during viral infection (84).

#### 3.4.1 DNA Viruses

It has been shown that Myc glycolytic targets are activated by the E4ORF1 protein of adenovirus to induce a Warburg effect-like status that converts glucose into nucleotides supporting replication of the virus (15). The Warburg effect has been reported in human and animal studies on viral infections as metabolism is signaled by higher glycolysis flow, rather than employing the more economical Krebs cycle for the production of energy (85). This results in increased lactate levels while TCA is kept in lower flow than in normal conditions (83). It is highly probable that reprogramming of the cellular genetic materials and protein translation machinery, as a result of the divergence and convergence of cellular and viral oncogenes targeting crucial protein mechanistic networks, is necessary for evolved pathological consequences (83).

Viral metabolomic studies were first adopted to characterize alterations in the host cellular metabolism caused by the HCMV infection of a human cell model (15, 85). These studies demonstrated that HCMV infection drives changes to specific metabolic signaling pathways that are necessary for the production and replication of the virus (15, 85).

Similar to carbohydrate metabolism, evidence on fatty-acid metabolic changes in the immune cells upon viral infections has been shown on many occasions and suggests an intermetabolic cooperation in glycolytic and fatty-acid pathways (86–89). HCMV also provides a model that crucially explores the characteristic features of sugar and lipid cellular cooperative metabolism to enhance its life cycle across species and how truncation of virus replication can be achieved by suppressing this cooperation (90). It has been further indicated that the shift in metabolism can eventually be an interest for the virus by helping the survival of infected cells (79). Still, targeting these modulated metabolic pathways can prevent the development of infection (85). For example, inhibition of the fatty-acid synthase (FAS) employing drugs such as C75 (trans-4-carboxy-5-octyl-3-methylene-butyrolactone) that shows specificity for HCMV infected cells or 5-(tetradecyloxy)-2-furoic acid (TOFA), which inhibits acetyl-CoA carboxylase enzymatic activity (85). It has also been proven that the inhibition of FAS has suppressed replication of many double-stranded viruses such as EBV (91) and VZV (92).

#### 3.4.2 RNA Viruses

Like DNA viruses, FAS repression has also demonstrated decreased virus replications in human immune cells such as in DENV (93), HCV (94), influenza virus (85), poliovirus (95), West Nile virus, and yellow fever virus (93). DENV infection has progressed to inflammation and metabolic abnormalities in mice and induced immune cell deterioration on the liver and spleen that were encountered upon stabilization of mast cells (96). Suggested attractive metabolic targets such as the glycolysis of the first enzyme hexokinase or glucose transporter 1 have shown upregulation following DENV infection (97). Viruses have different capabilities to adapt to each cell’s idiosyncratic environment, therefore, virus-induced metabolic programs are overall, cell type-specific (78, 98). Cells infected by RNA viruses such as HIV, HBV, and HCV have demonstrated alterations in the profile of their cellular metabolism (18, 99–101).

It becomes obvious that the metabolic components of the immune system, including innate and humoral cells, are crucial for the immune responses and can shift their behaviors based on either the specific viral invader or conditions in the human host (1, 83). HIV infection, for example, favors MQs and CD4^+^ T cells in which metabolism is promoted. The activation of divergent metabolic pathways shapes the metabolic environment of the cell and facilitates the viral replication cycle to support infection (101). It is a well-established dogma that CD4^+^ T cell activation is joined by the reprogramming of specific metabolic pathways, which includes a turn from quiescent cell oxidative metabolism to intensified aerobic glycolysis (92). Activated human monocytes extremely alter their metabolic phenotype from oxidative metabolism to the glycolytic pathway (102). Exceptionally, it was shown that oxidative metabolism was dominated, unlike the reduction in the uptake of glucose and glycolysis as the human U1 monocytic cell line was induced with HIV (103). An recent understanding of glucose metabolism and HIV infection was established, demonstrating that increased cellular permissiveness to HIV-1 infection was a result of CD4^+^ T cell increased expression of Glut1 in human cultures (104, 105). The process is partially mediated by the PI3K-Akt-mTOR cell survival pathway, which can be targeted in a therapy model in HIV-1 infection (99, 106).

Generally, the relationship between HIV pathogenesis in humans and immune cell metabolism is proposed as immune cells excessively uptake glucose, which could result in hyper induction of immune cells that progresses to complications of HIV infection (107). Still, the fact that our capacity to understand the exact influence of viral infection on the innate and humoral metabolism is limited to the little research effort done in this research area, what may not prevent us to hint on the specific differences between DNA and RNA viruses’ mechanisms to promote infection in the human host (108).

In another context, IFNs are potent modulators of the basic cellular processes (109). For example, PI3K/AKT-dependent uptake of glucose is stimulated by type I IFNs, which suggests an antiviral state as the inhibition of this glycolysis pathway enhances the replication of coxsackievirus B3 *in vitro* (110). Increased glycolysis is often accompanied by a hypoxia-inducible factor 1α independent, decreased oxidative consumption, a Warburg-effect state, which is as shown in experimental mice crucial to successfully prime CD4^+^ and CD8^+^ T cells (111). Type II IFN activation of MQ induces a break in the Krebs cycle flux compared to a high flow of glycolysis (110). This is accompanied by citrate and succinate accumulation (112). Citrate can drive the production of mitochondrial ROS, which holds a preserved role against a number of pathogens and apoptosis inducers (112, 113).

From these analyzed studies, it may appear that viral infections modulate the metabolic statuses in immune cells derived from either human or experimental animals, and a shift to a higher glycolytic activity is a common feature of this modulated metabolism (15, 82–86).

#### 3.4.3 COVID-19 Immunometabolism

##### 3.4.3.1 Immunometabolic Reprogramming During COVID-19

In the frame of a lethal circumstance such as SARS-CoV-2 viral infection, immune cells activate their metabolic reprogramming machinery to evoke this inflamed status and consequences. The ground of metabolic reprogramming during COVID-19 proposes a chance for metabolites with immunomodulatory properties to be interrogated as possible therapeutics to overcome the infection accompanied hyperinflammatory response (114). Using a murine model of SARS-CoV-2, human COVID-19 phenotypes have been mirrored by metabolic repression of oxidative phosphorylation and the TCA cycle in many organs with splenic atrophy, lymphopenia, and neutrophilia (115). One interesting finding in patients with severe COVID-19 is that monocytes/MQs circulated in the blood and are not involved in the generation of cytokine storm (116). It was explained that circulating DCs and monocytes/MQs in patients with severe COVID-19 are repressed by circulating tryptophan metabolites as a result of modulated immunometabolism (116).

On the other hand, the glycolytic shift into lactate has been evidenced by increasing disease severity showing an increase in lactate dehydrogenase activity in immune cells (116). Peripheral blood monocytes obtained from COVID-19 patients have demonstrated an increase in the respiratory mitochondrial electron transport chain (ETC) that has a crucial role in the generation of ROS and the cellular ATP molecules, which could propose a critical role in reprogramming immune cell metabolism to generate a critical immune response in COVID-19 patients (117, 118).

Obesity and factors related to it, such as increased fat intake and obesity-associated chronic inflammation as well as accompanied paradox attenuated innate immune response within the pulmonary compartment, may be crucial determinants in the host interaction with COVID-19 (119). Thus, adjustment of the immune cells’ metabolic potential and bioenergetics can centrally function in the innate immune response to this novel pathogen (119). In this concern, diet and nutrition may influence the outcome of COVID-19, while events such as patients with metabolic problems can change the whole infection progress (120).

In addition, age-related metabolic dysfunction in the innate immune cells due to alteration in glucose metabolism in the immune cells may predispose older individuals to severe outcomes during COVID-19 (121). To note, patients that are at a higher risk for the complications of the disease are usually diagnosed with other metabolic disorders, such as obesity and type II diabetes (122).

There is growing evidence is that some variants of coronavirus can evade immune reactions provoked by previous virus exposure and vaccination, raising attention and worry on some emerging mutations and variants that can manipulate normal metabolic status in immune cells (123).

##### 3.4.3.2 SARS-CoV-2 Mediated Immunomodulation’s Contribution to Mortality

**Figure 4:**
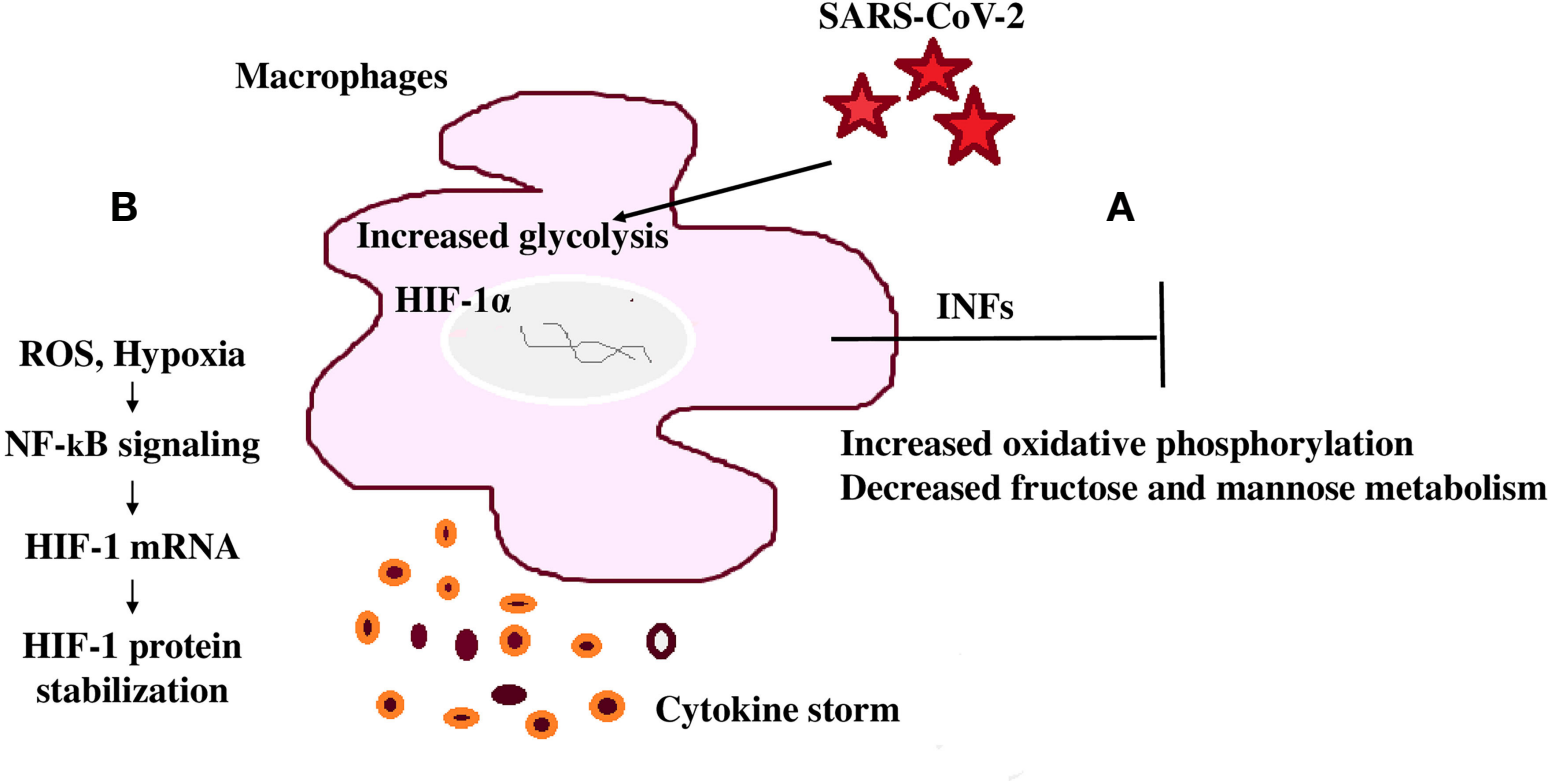
SARS-CoV-2 mediated immunomodulation contribution to mortality **(A)** COVID-19 patients share a common immunomodulatory failure to induce significant type I IFN responses in whole blood or PBMCs. This happens in concurrence with other modulated metabolic pathways including the upregulation of oxidative phosphorylation and downregulation of mannose and fructose metabolism. **(B)** SARS-CoV-2 spike protein induces monocytes and macrophages and increases glycolytic metabolism associated with the production of inflammatory cytokines. This response is dependent on hypoxia-inducible factor-1α (HIF-1α); SARS-CoV-2 infection triggers mitochondrial ROS production that stabilizes HIF-1α and consequently, activates glycolysis. HIF-1α-induced changes in monocyte metabolism directly inhibit T cell response that causes progression to epithelial cell COVID-19 mortality.

The lung is the initial infection site and lung failure is a severe complication of SARS-CoV-2 infection (124). Therefore, when evaluating clinical outcomes and mortality as a result of this infection, immune metabolic signatures in the lower respiratory tract of COVID-19 patients are important (124). A common immunomodulation feature observed in COVID-19 patients is the failure to induce significant type I IFN responses in whole blood or peripheral blood mononuclear cells (PBMCs) in concurrence with consistently modulated metabolic pathways including the upregulation of oxidative phosphorylation and downregulation of fructose and mannose metabolism and other glycan degradation (**Figure 4A**) (124). This leads to increased mortality because of the infection (124).

Monocytes undergo metabolic reprogramming and produce inflammatory cytokines when stimulated with SARS-CoV-2, highlighting the importance of this modulation in the induction of the cytokine storm (124). Stimulation of monocytes with recombinant SARS-CoV-2 spike protein subunit 1 showed a dose-dependent increase in glycolytic metabolism associated with the production of inflammatory cytokines (124, 125). This response is dependent on HIF-1α and evidenced through the inhibition of glycolysis and cytokine production by metformin or by 2-deoxyglucose or glucose deprivation (124). In this way, SARS-CoV-2 spike protein induces a pro-inflammatory immunometabolic response in monocytes that is linked to hyperinflammation during COVID-19 (124).

T cells play an essential and persisting role in the recovery and protection from acute SARS-CoV-2 infection (126). SARS-CoV-2 infection triggers mitochondrial ROS production, which induces stabilization of HIF-1α and consequently promotes glycolysis. HIF-1α-induced changes in monocyte metabolism directly inhibit T cell response and reduce epithelial cell survival progressing COVID-19 mortality (**Figure 4B**) (125).

It is noteworthy that monocyte-linked severe COVID-19 has been proven in human patients, as well as during experimental infections in different animal models (127–131).

## 4 Discussion

### 4.1 Diagnostics, Prognostics, and Biological Relevance of the Reviewed Findings

There is growing attention recently toward immunometabolism and its functionality in the frame of infection (1, 16). More precisely, the concept of manipulating glycolysis and oxidative phosphorylation significantly to help prevent disease progression to possibly affect innate and humoral immune cell sustainability and functionality (1, 16). This could result in the development of novel, tolerable immunotherapeutics in the future; early control of the infection or good prognosis of infection; or in the adoption of more preventive strategies based on our understanding of the pathogen/human metabolic mutual interaction (1, 12, 14, 16).

The interplay between host metabolism and bacteria, and the manipulation of the host metabolism by signaling pathways from these bacteria, or induced from the immune cells themselves or from the interaction of the immune cells and other pathogens, are quite of interest and suggests that either targeting or stimulating these signaling pathways can be of importance in fighting the bacterial pathogen (22, 24). Carbohydrate metabolism in the immune cells during TB is signaled by the glycolytic shift while with *P. aeruginosa* and *S. enterica* infections, activation is required of specific signals such as Glut1 or Akt1, which may help glucose transport that may in turn stimulate a glucose flux, suggesting that targeting the pathogens’ metabolism can be declared as a potential strategy for an effective remedy for human diseases of both bacterial infection and/or metabolic origin (36). The deep understanding of the challenge facing cell metabolism during infection by bacteria and how these metabolic burdens control the infection outcome is generally still limited. Challenges remain in this regard, in terms of the choice of suitable infection models or established cell lines or even the lack of consistency in the current methodological approaches as recently reviewed (46). It is widely evident that effector surface proteins are expressed on the surface of larger pathogens such as fungi and parasites as discussed using EPF and protozoa or helminths, respectively, to modulate metabolism in these cells, enhancing immune evasion by binding and activating host enzymatic cascades as well as the adhesion and binding of the pathogen to the extracellular matrix and basement membranes of the host cells (72). These proteins may not only be considered possible drug targets or vaccine candidates, but rather possible bioactive human medicines (65). Viral infections are able to demonstrate, beyond this hyper glycolytic activity, different strategies such as the inclusion of oncogenes, expression of modulatory metabolites, or the utilization of possible cooperation of different pathways (15, 84, 86, 90). Viral and cellular oncogenes can diverge and converge to develop critical networks of functional proteins that reprogram the cellular DNA and protein translational machinery, therefore targeting these oncogenes can be useful to prevent unwanted outcomes (12).

Taking into account that the metabolic reprogramming developed by viruses is cell-specific, according to the ability of each virus to compel host cells to adopt new distinctive settings (78). In this sense, targeting the modulatory metabolites or any cellular processes resulted from the interaction between different metabolic pathways for the sake of viral utilization or life cycle preservation can curtail virus replication specifically (85, 86, 90). IFNs are the potent modulators of basic cellular processes during viral infection (109, 110). Modulating, enhancing, or suppressing their signaling cascades may suggest an antiviral state (109).

## 5 Conclusions

A common feature during bacterial and viral infections is the upregulation of glycolysis signified by the lactate increase and disturbed TCA cycles signified by higher levels of succinate and other metabolically active intermediates such as itaconic acid. Furthermore, many glycolytic and TCA enzymes are detected on the surface of many fungi and parasites such as pyruvate carboxylase, pyruvate decarboxylase, SDH, and phosphoglycerate kinase, suggesting an unknown role of proteins and products secreted from these organisms in controlling carbohydrate metabolic cycles. Efforts taken to widen the understanding of both the metabolic new status of the pathogen-associated changes in carbohydrate metabolic pathways and signals involved in the pathogen–host interaction to determine specific pathogen signals and metabolites, would show future promise in terms of the design and usage of new vaccines or therapeutics and could help in building a new classification that can be used to adopt new strategies in diagnosis and treatment.

We may also conclude that SARS-CoV-2 can induce metabolic reprogramming in human immune cells and this could result to COVID-19 mortality in some patients; thus, metabolism in immune cells can possibly serve as a potential therapeutic approach to target COVID-19 in a cell-specific manner.

## Data Availability Statement

The original contributions presented in the study are included in the article/supplementary material. Further inquiries can be directed to the corresponding authors.

## Author Contributions

MB conceptualized, structured, and edited the manuscript. KA gathered scientific materials, and analyzed and wrote the manuscript. AM edited and contributed to the immune-metabolism in bacteria section. DA-E edited and contributed to the section on viruses’ immunometabolism. All authors revised and approved the final manuscript.

## Conflict of Interest

The authors declare that the research was conducted in the absence of any commercial or financial relationships that could be construed as a potential conflict of interest.

## Publisher’s Note

All claims expressed in this article are solely those of the authors and do not necessarily represent those of their affiliated organizations, or those of the publisher, the editors and the reviewers. Any product that may be evaluated in this article, or claim that may be made by its manufacturer, is not guaranteed or endorsed by the publisher.
